# Accurate Measurement of Magnetic Resonance Imaging Gradient Characteristics

**DOI:** 10.3390/ma7010001

**Published:** 2013-12-19

**Authors:** Hui Liu, Gerald B. Matson

**Affiliations:** 1 Center for Imaging of Neurodegenerative Diseases, Veterans Affairs Medical Center, San Francisco, CA 94121, USA; E-Mail: maggiehuil@yahoo.com; 2 Department of Pharmaceutical Chemistry, University of California, San Francisco, CA 94143, USA

**Keywords:** magnetic resonance imaging (MRI), gradients, impulse response function

## Abstract

Recently, gradient performance and fidelity has become of increasing interest, as the fidelity of the magnetic resonance (MR) image is somewhat dependent on the fidelity of the gradient system. In particular, for high fidelity non-Cartesian imaging, due to non-fidelity of the gradient system, it becomes necessary to know the actual k-space trajectory as opposed to the requested trajectory. In this work we show that, by considering the gradient system as a linear time-invariant system, the gradient impulse response function (GIRF) can be reliably measured to a relatively high degree of accuracy with a simple setup, using a small phantom and a series of simple experiments. It is shown experimentally that the resulting GIRF is able to predict actual gradient performance with a high degree of accuracy. The method captures not only the frequency response but also gradient timing errors and artifacts due to mechanical vibrations of the gradient system. Some discussion is provided comparing the method presented here with other analogous methods, along with limitations of these methods.

## 1. Introduction

### 1.1. Overview

In modern magnetic resonance imaging (MRI) instruments, the fidelity of the MR image is dependent upon the fidelity of the gradient system, such that the actual gradient outputs closely match the requested outputs. For conventional imaging, in which k-space points on a Cartesian grid are acquired, the image fidelity is somewhat immune to small gradient discrepancies, as long as the gradient areas are largely preserved. On the other hand, more efficient MRI data collection schemes that do not acquire the data on a Cartesian grid, such as spiral imaging, are sensitive to even small discrepancies in the gradient waveforms, which can give rise to significant blurring in the resulting images [1].

As there has been increased interest in recent years in more efficient data collection efficiencies for MRI, in which the data are not collected on a Cartesian grid, there has been a corresponding increased interest in methods for measuring the actual gradient output, with the purpose of providing corrections to improve the fidelity of the resulting image. For the most part, the gradient outputs are monitored by measuring the k-space locations actually acquired, as opposed to the k-space locations that would have been acquired by gradients of perfect fidelity.

There are multiple sources giving rise to gradient infidelity, including: non-linear gradient amplifier amplification and limited gradient amplifier frequency response, incomplete eddy current compensation, including gradient cross terms, the existence of a non-linear gradient field itself, and so-called concomitant gradients, which are higher order spatially varying magnetic fields which necessarily accompany the desired linear gradient fields [2].

### 1.2. Previous Work

For the most part, early approaches for measuring the gradient fields in MRI instruments (either to adjust eddy currents, or to measure k-space trajectories) involved measurements with small phantoms [3–6] These early approaches have been followed by more efficient techniques based on imaging methods in which the phantom is replaced by a selected slice [7–11], and signal reception is accomplished by the imaging coil. One limitation of these methods has been that, to accurately measure the k-space trajectory to be used, the width of the slice must be on the order of, or smaller, than the spatial resolution to be used in the image reconstruction. This limits the S/N, and to some extent the accuracy with which the k-space trajectory can be measured [12]. A more complete review of these methods, together with a brief discussion of their strengths and weaknesses, is provided by Han *et al.* [12].

Instead of measuring the actual k-space trajectory, it is possible to consider the gradient system as a linear, time invariant system and measure the gradient impulse response function (GIRF). As discussed in detail in the Theoretical Background section, the actual gradient waveform is then predicted by applying the GIRF to the requested gradient waveform. The idea of considering the gradient system as a linear, time invariant system and obtaining a measurement of the GIRF to predict gradient response is not new, and has been presented in some detail by, for example, Alley *et al.* [10], who used an imaging technique to obtain the GIRF measurements. To some extent, the accuracy of spiral k-space trajectories can be improved by proper adjustment of gradient delays [1]. However, the conclusion by Addy *et al.* [13] was that, at least for correction of spiral trajectories, the GIRF method was superior to just adjusting gradient delay times. Recently, the Pruessmann group [14–16], has proposed an elegant, although experimentally difficult, approach to measuring gradient fields with unprecedented accuracy. Some of the complexities arise due to one of their initial primary goals: To devise an approach that enables the gradients to be monitored during the actual MR imaging experiment. Their approach involves the construction of multiple, small samples (on the order of 1 mm diameter), each with their own receive coil and receive circuit, and susceptibility matched, so that the signal of each sample can be followed throughout the duration of a gradient waveform to be used in the MR imaging sequence. In analogy with imaging experiments to measure the k-space trajectory, the sample size, and thus the S/N of a single measurement, is limited by the resolution to be obtained by the k-space trajectory to be measured. Their approach, combined with the ability to use multiple receive channels to accommodate simultaneous reception from the multiple samples, has been demonstrated to provide an efficient means for monitoring k-space trajectories of interest [16].

To alleviate the difficult susceptibility matched sample and probe construction utilized by the Pruessmann group, the Balcom group [12,17] has emphasized the use of a slightly larger, more heavily doped phantom, to be used with a train of radio frequency (RF) pulses and resulting phase measurements, reminiscent of the method suggested by Wysong and Lowe [3] for measurement (and adjustment) of gradient eddy currents. The strength of this approach is, because the sample is excited repeatedly, the approach is applicable to gradients of long duration and the sample size can be larger than that dictated by the Pruessmann group approach. Because the sample must be excited during application of the gradient, the signal phase induced by the off-resonance pulses must be addressed. However, as long as the pulse length is short enough, and the pulse length and tip are known, this can readily be accounted for. Alternatively, multiple, overlapped measurements may be taken with the phase of the signal adjusted to match the previous overlapped segment. However, a further, potential difficulty with their approach is that many commercial MRI instruments may not accommodate a rapid sequence of pulses, each followed by a short delay and signal measurement.

## 2. Results and Discussion

### 2.1. Theoretical Background

A number of articles have shown that, over limited gradient strengths and field of views, and within manufacturer prescribed slew rates and duty cycles, to a relatively high degree of accuracy the gradient response can be considered as a linear, time invariant system [10,13,16,18]. Thus, in this situation the gradient output, *out*(*t*), is related to the desired waveform, *in*(*t*), according to the impulse response *h*(*t*) [16]:

$$out(t)=\overset{\infty }{\underset{-\infty }{\int }}in(\tau )\times h(t-\tau )\text{d}\tau$$ (1)

Taking the Fourier transform, such that:

H(ω)=FT(h(t)),andIN(ω)=FT(in(t)) (2)

The relationship may be expressed as:

OUT(ω)=IN(ω)×H(ω) (3)

Vannesjo *et al.* [16] label the function *H*(ω) the gradient impulse response function (GIRF). Through signal averaging, they have demonstrated the ability to measure the gradient system GIRFs with unprecedented accuracy. A particular advantage of this approach is that it is independent of any models of eddy current behavior, and in addition intrinsically includes effects due to gradient mechanical vibrations. In this article, we limit ourselves to the situation in which Equation (3) applies, although we briefly touch on non-linear effects in the Discussion section.

Our approach can be thought of as something in between the Pruessmann and Balcom approaches. Adopting some of the Balcom ideas, we use a single, small phantom, with its own transmit/receive coil. As the phantom size is several mm in diameter and not susceptibility matched, the construction is relatively straightforward. The phantom has its *T*_1_ reduced to approximately 100 ms to enable fairly rapid pulsing on the sample. Unlike the Balcom approach, for the GIRF determination we do not apply RF pulses while the gradient is on, and more like the Pruessmann approach, we do multiple signal sampling during the free induction decay (FID) to follow the phase evolution of the sample signal. Overlapping acquisitions are used to enable sampling over the desired time period. Although Addy *et al.* [13] use a chirp gradient waveform to determine their GIRF function, we follow the Pruessmann approach [16] and use a series of triangular waveforms for mapping the frequency response of the gradient system. This has the added advantage that additional signal averaging can be used on the short gradient waveforms (which emphasize the higher frequency response of the gradient system) where the S/N is inherently lower than results from the longer waveforms.

Following a more complete description of our method, we demonstrate that our measured GIRFs are highly accurate and able to accurately predict measured gradient waveforms. Moreover, our GIRFs can be expected to provide important corrections to non-Cartesian gradient trajectories such as spiral gradient waveforms. The primary advantage of our method is that the ease of implementation of our approach should facilitate duplication by other MRI labs, and the experiments are readily implemented on a commercial MRI system.

### 2.2. GIRF Calculations

As indicated in the Background, the GIRF measurements involve comparing the gradient waveform actually produced with the requested waveform. The actual gradient waveform is obtained from differentiation of the sample signal at a known location. That is, as the frequency Ω is given by:

$$\Omega (x)=\gamma xGx=\frac{d\varphi }{dt}$$ (4)

We have:

$$Gx=\frac{1}{\gamma x}\frac{d\varphi }{dt}$$ (5)

where γ is the gyromagnetic ratio, *x* is the distance from isocenter, *G* is the gradient along the *x* dimension, and φ is the phase of the signal.

While Addy *et al.* [13] compared using a gradient chirp waveform to a single triangular waveform and found the chirp method to produce more accurate results, the Vannesjo *et al.* [16] approach used a series of triangular waveforms of identical gradient slew rates to generate response functions covering the frequency response of the gradient system. One advantage of this later approach is that more signal averaging can be used with the shorter waveforms to improve the S/N of the higher frequency regime of the GIRF. As shown by Vannesjo *et al.*, a triangular input function corresponds to a squared sinc function:

$$IN(\omega )\propto s\frac{sin2(\omega T/2)}{\omega 2}$$ (6)

where *s* is the slope of the gradient of length 2*T*. Finally, these authors show that a reasonable way to combine the measurements from triangular waveforms of the same slope but different lengths to obtain an estimate of the GIRF *H*(ω)can be written as:

$$\hat{H}l,m(\omega )=\frac{\sum_{j}IN_{l,j}^{*}(\omega )\cdot OUT_{m,j}^{l}(\omega )}{\sum_{j}{\left|IN_{l,j}(\omega )\right|}^{2}}$$ (7)

where *l* represents the input channel, *m* the output channel, and *j* a particular triangular waveform. To keep the notation simple, we use GIRF*xx* for ${\hat{H}}_{x,\;x}(\omega )$, *etc.*

**Figure 1.**
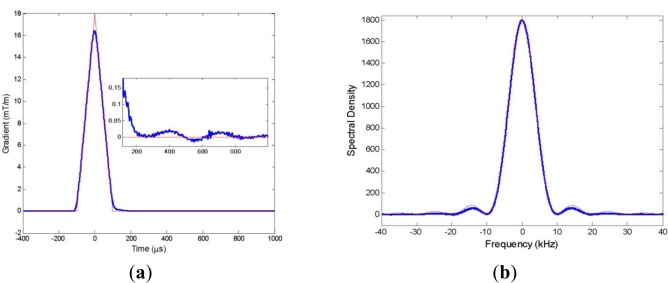
(**a**) The requested triangular gradient waveform in the time domain (red), and the measured gradient (blue). The inset shows that some residual gradient persists for up to several ms beyond the nominal end of the gradient; (**b**) The requested triangular gradient waveform in the frequency domain (red) and the measured gradient waveform (blue). The figure shows that the higher frequency components (above 10 kHz) are diminished in the measured waveform as compared to the requested waveform.

The calculations of the GIRFs were done as indicated by Equation (7). Figure 1a shows an example of a triangular gradient input signal of risetime length *T* of 100 μs (red), and the resulting output gradient (blue). On all channels, the gradients returned to zero amplitude (to within the accuracy of our measurements) within a few ms. The same input and output signals in the frequency domain are shown in Figure 1b. The discrepancy above 10 kHz is clearly shown. However, the frequency content of even this short gradient pulse above 20 kHz is quite low.

**Figure 2.**
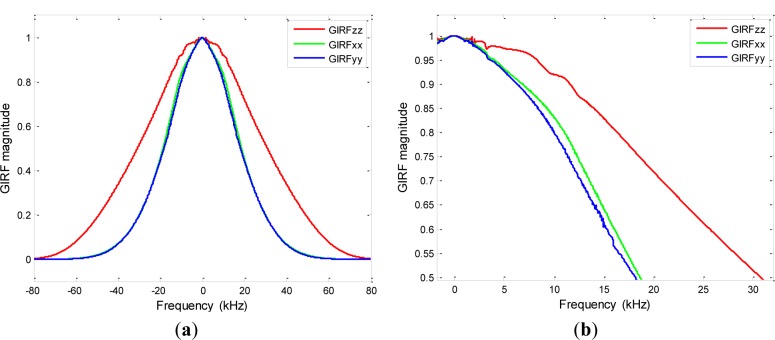

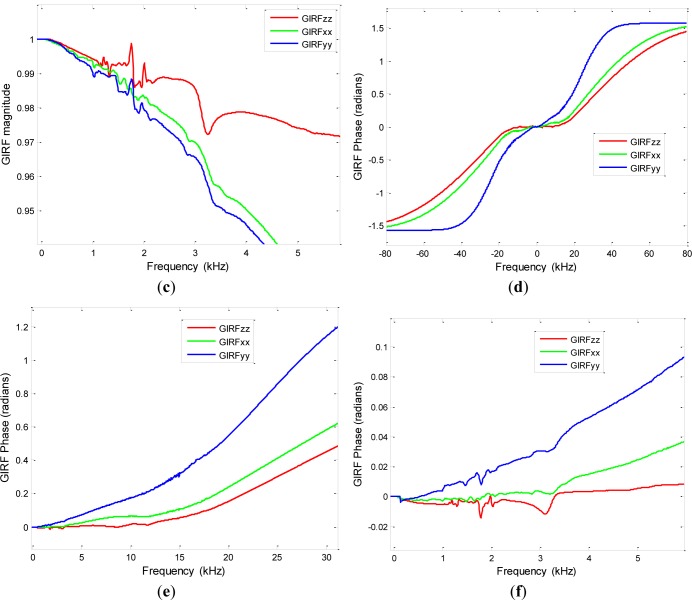
Plots of gradient impulse response function (GIRF) magnitude and phase for the three gradient directions. (**a**–**c**) Increasingly zoomed plots of GIRF magnitude. The overall frequency response is apparent in (**a**), while more detail, including mechanical resonances are apparent in (**b**) and (**c**). While the magnitude GIRFs for the *x* and *y* gradients closely follow each other, the *z* gradient exhibits a rather higher frequency response. (**d**–**f**) Increasingly zoomed plots of GIRF phase. While the *z* and *x* gradient phases exhibit similar changes with frequency, the *y* gradient shows a more rapid phase change. The more zoomed plots (**e**) and (**f**) show the same mechanical resonances as seen in the magnitude plots. Small artifacts very close to zero frequency in (**f**) are due to the data processing.

Plots of magnitude GIRFs in the frequency domain are shown in Figure 2a–c. GIRF cross responses (GIRF*xy*, *etc.*) were also measured, along with B_0_ terms. However, as these responses were all below 0.1%, they are not displayed here. Somewhat surprisingly, the GIRF*zz* magnitude plots showed a much broader frequency response than the *x* and *y* GIRFs. The extrapolated *z* GIRF magnitude goes to zero just past 70 kHz, while the *x* and *y* GIRF magnitudes go to zero just past 50 kHz. This suggests a much lower inductance and/or smaller resistance in the *z*-gradient windings compare to the *x*- and *y*-gradient windings. On the other hand, the *x* and *y* GIRFs tracked each other fairly closely. Several sharp frequencies are apparent in Figure 2c, at frequencies from just over 1 kHz to just over 2 kHz, and are ascribed to mechanical vibrations (acoustical resonances). Further, broader frequency responses are apparent at around 3 kHz for the *x* and *y* gradients, and just beyond 3 kHz for the *z* gradient. The origin of these broader responses is not known.

The phases of the GIRFs are shown in Figure 2d–f. While the phase from the *x* and *z* gradients are similar, the *y* gradient shows a more rapid phase response. Figure 2e,f show the phase of the *x* and *y* GIRFs are close to zero out to several kHz, while the *y* GIRF deviates from zero beyond 1 or 2 kHz. Together, these phase plots suggest that while the gradient delays are correct for the *x* and *z* gradients, the delay for the *y* gradient could be improved. The phase plots do show some small irregularities very close to zero frequency, presumed to be an artifact as a result of eliminating the small spike at zero frequency on the GIRF magnitude plot. Finally, the same sharp frequency responses apparent in the magnitude plots are reproduced in the same frequency positions in the phase plots.

**Figure 3.**
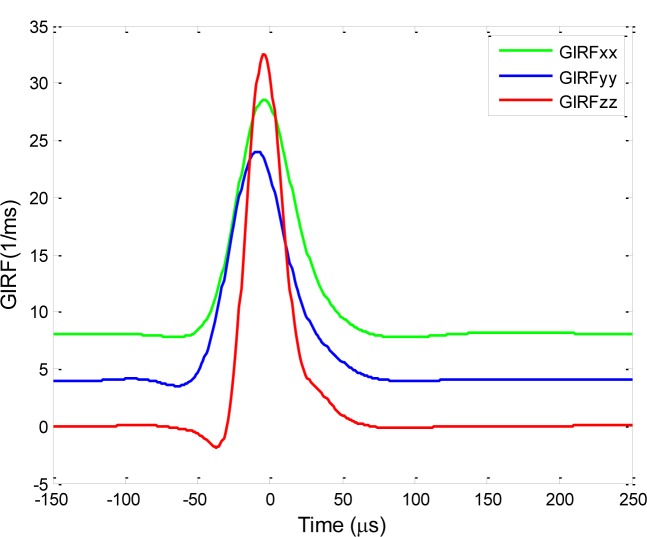
Time domain plots of the GIRFs in the three gradient directions. The plots are offset in the vertical direction for clarity. All plots show amplitudes prior to zero time. The higher frequency response of the *z* gradient results in a narrower GIRF in the time domain. While the peak amplitudes of the *x* and *z* GIRFs occur close to zero time, the *y* GIRF peak amplitude occurs at slightly negative time.

Figure 3 shows the GIRFs in the time domain, where the GIRFs are offset in *y* to better enable them to be distinguished from one another. The broader frequency response of the *z*-gradient results in a considerably narrower and higher amplitude time response for this GIRF. While all three time response GIRFs have amplitudes prior to zero time (reflecting the fact that the correct gradient delays must account for the less than perfect frequency response of the gradient amplifiers). However, the peak of the *y* GIRF occurs at slightly negative time, again suggesting a somewhat incorrect delay for this gradient. Finally, it is apparent that the GIRFs rise times are shorter than the fall times, with all GIRFs exhibiting slight negative amplitudes just prior to their rise.

### 2.3. GIRF Predictions

Assuming the gradient systems to be linear and time invariant enables the actual gradient shape produced in the frequency domain to be predicted by Equation (3). Inverse Fourier transform then produces the predicted gradient in the time domain. To demonstrate the ability of the GIRF approach to predict the actual gradient shape, we produced a trapezoidal gradient shapes with identical rise, top and fall times, with 180 mT/m/ms slew.

**Figure 4.**
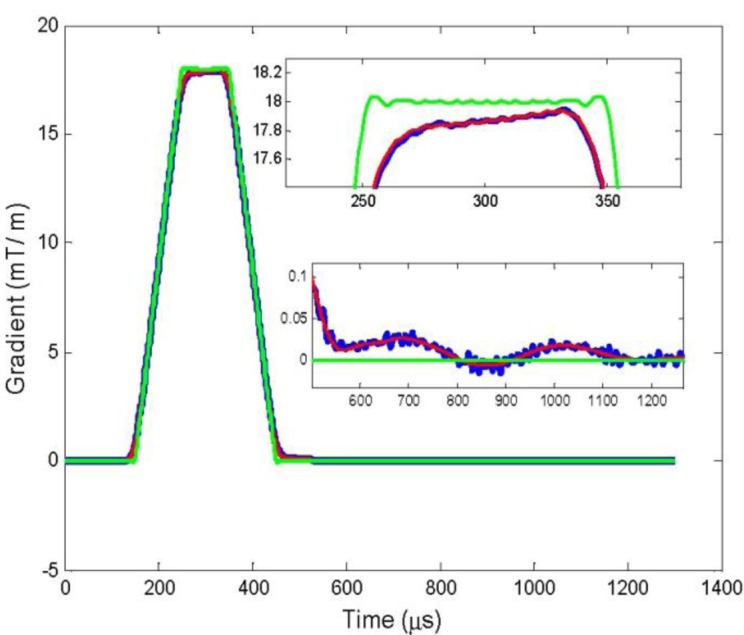
Requested (green), predicted (red) and experimental trapezoidal gradient waveforms for a *y* gradient with 100 μs rise, top, and fall times, and slew rate of 180 mT/m/ms. The zoomed insets show significant discrepancies between the requested and experimentally measured gradient. Despite the noise on the experimental gradient measurement, it is clear that the GIRF prediction provides a highly accurate description of the actual gradient waveform.

Figure 4 shows the results for an *x*-gradient trapezoidal pulse with rise, top, and fall time of 100 μs, where the pulse was calculated from a data set of 200 averages with TR of 500 ms and a sweep width of 100 kHz. The requested gradient shape is shown in green, the measured gradient in blue, and the GIRF prediction in red. The zoomed insets on the figure show the gradient top and region just following the end of the gradient in greater detail. Despite significant noise on the experimental result, the figure demonstrates the GIRF prediction accurately predicts the actual gradient shape to a high degree of precision. Interestingly, while the output shows clear rounding of the corners, the gradient area agrees with that of the input to within 0.03%. Although we present only a single result here, similar results were obtained from trapezoidal gradients on the other gradient axes.

### 2.4. Discussion

As outlined in the Introduction, quite a variety of imaging methods have been developed, for purposes of adjusting eddy currents, measuring k-space trajectories, or obtaining GIRFs. Some recent, rather sophisticated approaches include an elaborate imaging method for obtaining variable timing delays for improving spiral k-space trajectories [19], the use of chirp gradient waveforms to obtain the frequency response in a single experiment [13,20], an array of multiple phantoms of different chemicals for obtaining simultaneous, distinguishable signals for eddy current measurement and compensation [21], and the multiple, small phantom arrays, each phantom with its own transmit/receiver system, used by the Pruessmann group [16], which enable field measurements over different spatial positions in a single experiment. The results of Vannesjo *et al*. [16] are particularly notable in that their high precision GIRF measurements of the gradient system response include effects due to gradient mechanical vibrations. Furthermore, they show that their GIRF measurements enable accurate predictions of the gradients produced. However, duplication of their elaborate, sophisticated, small phantom (1 mm diameter) array, each phantom with its individual transmit/receive coil, is probably beyond the capability of most MRI laboratories.

Influenced in part by the reasoning and approach suggested by the Balcom group [17], we have elected to perform the GIRF measurements with a rather simple single coil and phantom system, and used the simple triangular gradient waveforms suggested by Vannesjo *et al*. [16] for the GIRF measurements. While our larger size phantom provided increased S/N as compared to the small Vannesjo *et al*.[16] phantoms, we still found it necessary to perform some signal averaging to obtain high quality GIRF measurements. The primary disadvantage of our approach is that the sample must be moved to measure the GIRFs along the different Cartesian axes, and measurements of the gradient fields in terms of spherical harmonics as done by Vannesjo *et al*. [16] would require measurements over a rather large number of sample positions. However, the simple sample and coil construction, and simple pulse programming necessary for producing the gradient triangular waveforms, could be readily performed by many labs.

Had we taken only the 100 kHz data (See Experimental Section), the time to perform our GIRF measurements along one axis (with 400 averages and nine gradient waveforms) would have been one hour. Thus, the primary disadvantage of our approach is the time required for the measurements. This makes it difficult, for example, to see if the GIRFs are altered with gradient temperature, although such measurements could still be made by alternating GIRF measurements with MRI sequences to maintain the gradients at some elevated temperature.

Another potential disadvantage of our use of a larger phantom is that longer time duration gradient waveforms may require interleaved experiments to make experimental measurements of the waveform. On the other hand, the larger size phantom does improve the initial S/N of the experiment.

The GIRFs may be utilized in a variety of ways to improve image fidelity, particularly in non-Cartesian imaging. For example, Addy *et al*. [13] outline the use of GIRFs for improved fidelity in image reconstruction, and our own motivation for GIRF measurement stems from our interest is spiral imaging with variable density spiral gradient waveforms. Even in Cartesian imaging, knowledge of the GIRFs would enable pre-warping of the gradient input waveform to improve gradient fidelity near the edge of flat top gradient waveforms, and improve the turnoff performance. For example, note the slope of the top of the gradient waveform in Figure 6, and the existence of small gradient effects persisting following turn off of the gradients. While the frequency response of the system cannot be improved, both fidelity and gradient moment could potentially be improved through pre-warping [22].

Finally, the assumption of linearity and time invariance does have limitations. For example, gradient heating in gradient intensive MRI sequences may alter the GIRF, and the assumption of linearity means that concomitant gradients cannot be taken into account by the GIRF approach. In addition, the GIRF is not valid if the maximum slew rate is exceeded. Finally, the spatial non-linearity of the gradients must be accounted separately. For this reason, we used small displacements from isocenter (approximately 50 mm) to ensure we were well within the region of spatial linearity of the gradient system.

## 3. Experimental Section

### 3.1. Phantom and Coil

**Figure 5.**
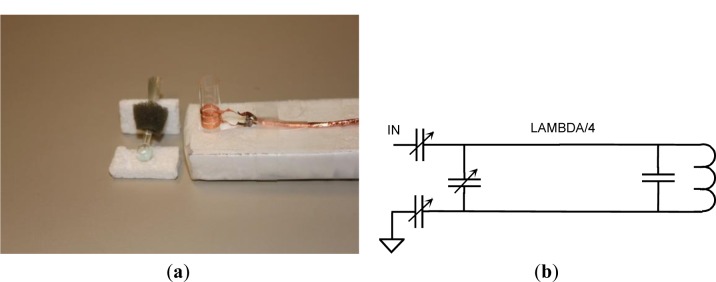
(**a**) Photograph of the sample and coil used for the GIRF experiments. The coil is wound around an 8 mm NMR tube; (**b**) Diagram of the coil and capacitor, with the adjustable tuning capacitors situated a quarter wavelength away from the coil.

As a compromise between small size and ease of construction, we chose a Wilmad 529-A-8 spherical bulb insert, with a volume of approximately 110 μL, which fits inside an 8 mm NMR tube. A foil coil winding was formed around the NMR tube as shown in Figure 5a. The coil was tuned with a chip capacitor at the coil to just over the 3.0 Tesla Larmor resonance frequency of 123 MHz, with the adjustable capacitors for fine tuning placed one quarter wavelength away (Figure 5b) to minimize any influence of the adjustable capacitors. The slightly higher tuned frequency of the capacitor/coil system is seen as capacitive impedance at the Larmor frequency, which is reflected back as inductive impedance at the end of the quarter wavelength line, where it can be tuned to 50 ohms resistive by standard means.

The quarter wavelength cable used a Teflon dielectric, as previous experience showed significant contamination signal arose from use of a cable with polyethylene dielectric. Signal loss from use of the quarter wavelength cable was minimized by adjusting the coil and chip capacitor tuning close to the MRI resonance frequency. The components were mounted on a polystyrene foam board. The sample was doped with copper chloride solution to obtain a *T*_1_ of around 100 ms. The signal-to-noise ratio (S/N) at a bandwidth of 100 kHz approached 1000. Using ξ as the sensitivity measure, defined as the S/N multiplied by the bandwidth (BW) [14]:

$$\xi =\text{S}/\text{N}\sqrt{BW}$$ (8)

yields a ξ of around 3 × 10^5^. To connect the coil to the Siemens Skyra MRI system, an interface box and coil file (software file that Siemens requires as part of interfacing the coil to the MRI instrument) were purchased from Stark Contrast (See Acknowledgments).

### 3.2. Experimental Measurements

**Figure 6.**
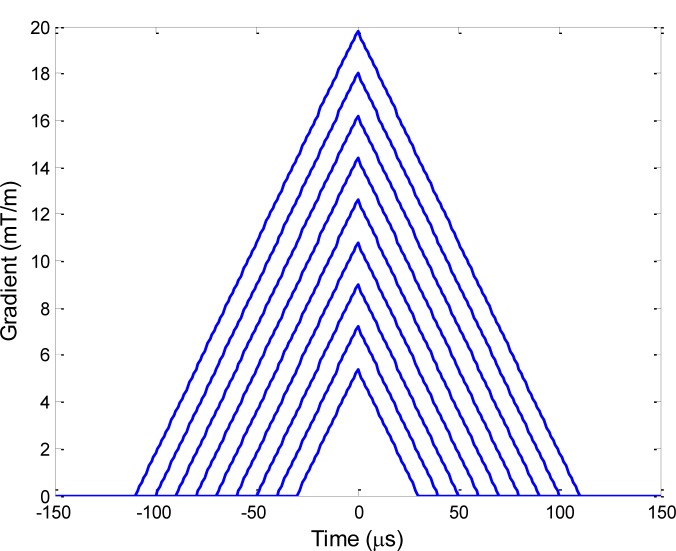
The series of triangular gradient waveforms use for the GIRF calculations. The rise and fall times varied from 30 μs to 110 μs in increments of 10 μs, and all waveforms used a slew rate of 180 mT/m.

For most of our measurements on our Siemens Skyra 3.0 T system with XQ gradients, we used a series of nine triangular gradients of slew rate 180 mT/m/ms, and rise and fall times T in increments of 10 μs from 30 μs to 110 μs as illustrated in Figure 6. As the Skyra system only allowed a maximum of 4096 data points to be collected on a single measurement, results at bandwidths of 100 kHz and 25 kHz were concatenated, with oversampling at factor of 2, to provide data collections durations of 20.48 and 83.92 ms. All data were collected by interleaved sets of alternating gradient on and gradient off, with TR of 500 ms. For the 100 kHz bandwidth acquisitions 400 data sets were acquired, while 200 sets were acquired for the 25 kHz data sets. For the GIRF calculations, the background data were collected as the average of the gradient off data prior to and following the gradient on data.

The data sets for the GIRF calculations were oversampled to 1 μs dwell times, and spliced together so that the initial data length of 20.48 ms was covered by the larger bandwidth signal, and the rest by the lower bandwidth signal. The rational for this was that the residual gradients at longer times arose primarily from incomplete cancellation of the longer time constant eddy currents, whose frequency content was adequately covered by the lower bandwidth acquisition.

To improve the S/N, particularly in the high frequency regime of the GIRF, Savitzky-Golay filtering was done, with greater smoothing performed on the higher frequency region of the GIRF. For a variety of reasons, the GIRF calculations became unreliable beyond approximately 30 kHz, and even showed an artificial upswing in the very high frequency regime of the GIRF. To avoid this artificial upswing, a two term polynomial was fitted to a logarithm of the GIRF over the 16 kHz to 27 kHz region, and used to extrapolate the GIRF to zero. The same procedure was performed on the magnitude and real GIRFs, and the imaginary GIRFs calculated to be consistent with the difference between magnitude and real components. As actual requested gradients in general do not have significant frequency component at these high frequencies (above 20 kHz), this did not result in significant errors in application of the GIRFs to calculate resulting gradients from requested waveforms. In addition, some of the calculated GIRFs exhibited a small blip at zero frequency, presumably to slight instrument drifts that were not completely cancelled out. These blips at zero frequency were removed to provide more realistic estimate of the GIRFs.

Finally, the phantom was positioned at approximately 50 mm from isocenter along the axis being measured for the GIRF measurements (and at isocenter for B_0_ measurements). The initial estimates of the phantom position were obtained by measuring the frequency in the presence of a 0.5 mT/m gradient. However, due to somewhat asymmetric lineshapes, there remained some small uncertainty in the exact position. Therefore, a slight adjustment was done on the phantom positions so that the GIRF*xx*, GIRF*yy*, and GIRF*zz* calculations were unity at zero frequency. This amounted to assuming that the MRI engineers had adjusted the gradient strengths correctly.

In addition to the GIRF measurements, gradient measurements of additional trapezoidal gradient waveforms with equal rise, top, and fall times were taken to compare with the GIRF predictions of these waveforms. The gradient shapes were measured using the identical setup as used for the GIRF calculations, with the phantom positioned approximately 50 mm from isocenter. As done with the GIRF calculations, repetitions were taken with alternating gradient and background acquisitions. As before, the background data were collected as the average of the gradient off data prior to and following the gradient on data, and the actual gradient calculated by Equation (5).

Some additional experiments were performed at still higher bandwidths (500 kHz), but these acquisitions were not found to improve the GIRF estimations. In addition, some low bandwidth acquisitions (at 25 kHz) were done with a sample with longer *T*_1_ and *T*_2_^*^ to obtain a longer time acquisition, as the sample with 100 ms *T*_1_ (and somewhat shorter *T*_2_^*^) did not produce reliable results beyond approximately 50 ms. However, these longer time acquisitions also did not improve the GIRF estimations, and there was no evidence of persistence of gradient effects beyond several ms after turnoff of the gradients. Thus, it appears that acquisitions using just the 100 kHz bandwidth acquisitions would have given reasonable GIRF estimates. For this reason, in the Discussion section we discuss the time for the experiments as if we had just taken the 100 kHz bandwidth measurements.

## 4. Conclusions

We have presented a simple approach for obtaining GIRFs with relatively high precision, and shown that this approach does provide accurate predictions of actual gradient waveforms. Our measured GIRFs capture both the frequency response of the gradient amplifier, as well as deviations due to mechanical vibrations (and potentially other sources as well). Our approach utilizes a rather simple sample and coil construction, and simple MRI experiments with triangular gradient shapes to enable GIRF calculations. Other than the restrictions imposed by the assumption of linearity and time invariance, the primary disadvantage of our approach is the time required to make the measurements.
